# The Role of Physicians’ Digital Tools in Pharmacological Management of Type 2 Diabetes Mellitus

**DOI:** 10.3390/medicina58081061

**Published:** 2022-08-06

**Authors:** Andrej Janež, Rok Ješe, Martin Haluzík, Manfredi Rizzo

**Affiliations:** 1 Department of Endocrinology, Diabetes and Metabolic Disease, University Medical Centre Ljubljana, Zaloska 7, 1000 Ljubljana, Slovenia; 2 Faculty of Medicine, University of Ljubljana, Vrazov trg 2, 1000 Ljubljana, Slovenia; 3 Diabetes Centre, Institute for Clinical and Experimental Medicine, Videnska 1958/9, 140 21 Praha, Czech Republic; 4 Department of Health Promotion Sciences Maternal and Infantile Care, Internal Medicine and Medical Specialties (PROMISE), School of Medicine, University of Palermo, 90100 Palermo, Italy

**Keywords:** type 2 diabetes, digital tools, personalized treatment, GLP-1, DPP4, SGLT2

## Abstract

*Background and Objectives*: The constantly increasing prevalence of type 2 diabetes mellitus (T2DM) and the advent of new treatment options have made management of T2DM patients more demanding. We aimed to (a) estimate the familiarity of general practitioners with novel T2DM treatment options, (b) determine whether a digital tool can aid in their treatment decisions and (c) demonstrate that an evidence-based digital clinical support tool can be made using an existing digital platform. *Materials and methods*: This proof-of-concept study consisted of two parts: We first conducted a simple online survey among general practitioners of three European countries to estimate their familiarity with novel T2DM treatment options and to determine whether they believe that a digital tool can aid in their T2DM treatment decisions. We then proceeded to develop a new digital tool that provides quick, evidence-based support for treatment of patients with T2DM using an existing digital platform. *Results*: The online survey was completed by 129/5278 physicians (94 from Italy, 22 from Czech Republic and 13 from Slovenia). Only 30.7% of all general practitioners reported to be either very or extremely familiar with novel T2DM treatments; the vast majority of participating general practitioners (82.8%) reported that they would find a digital clinical decision support tool for treating T2DM patients either very or extremely useful. A digital tool which features the characteristics deemed most important by the polled physicians was subsequently developed. *Conclusions*: The results of the online survey showed that familiarity of general practitioners with novel T2DM treatment options is relatively low and that there is a need for digital clinical decision support tools intended to facilitate treatment decisions in T2DM patients. We demonstrated that such a tool can easily be developed using an existing digital platform.

## 1. Introduction

Type 2 diabetes mellitus (T2DM) is an expanding global health problem, characterized by dysregulation of carbohydrate, lipid, and protein metabolism. Insulin resistance, one of the hallmarks of T2DM, is closely associated with obesity. T2DM also carries a highly elevated risk of both microvascular complications (such as diabetic retinopathy, neuropathy, and nephropathy) and macrovascular complications (such as coronary artery disease, cerebrovascular disease, and peripheral artery disease) [1].

The rapid evolution of our understanding of T2DM has expedited the development of novel medications and several new antidiabetic medications have gained widespread use in the last decade, including dipeptidyl peptidase-4 (DPP-4) inhibitors, glucagon-like peptide-1 (GLP-1) receptor agonists, and sodium/glucose cotransporter 2 (SGLT2) inhibitors, which have been also incorporated in the latest international scientific guidelines for the management and treatment of T2DM [2,3,4,5]. Since it is currently not possible to reverse the multiple pathophysiological abnormalities present in T2DM patients using only one drug, combination therapy has also gained widespread acceptance. Additionally, some of the novel medications have demonstrated significant benefits for patients with prevalent comorbidities, such as atherosclerotic cardiovascular disease, heart failure, and chronic kidney disease [6,7].

The constantly increasing prevalence of T2DM worldwide has made management of T2DM more demanding [1]; in order not to needlessly burden the practicing healthcare professionals with information overload, the ability of communicating the most recent recommendations in a clear and concise manner has become paramount. This is further complicated by the fact that the use of anti-diabetic medications with proven cardiovascular benefit is sub-optimal worldwide, although clinicians are fully aware that their T2DM patients most likely suffer and may sadly die for cardiovascular complications [8]. A recent report from experts working in Europe’s east and south countries has shown that many barriers exist for the implementation of the international scientific guidelines and that new measures are awaited to facilitate their implementation [9]; this again emphasizes the urgent need of filling the gap between guidelines and real world for proper management of T2DM patients [10].

Fortunately, these challenges are paralleled by the digital evolution. Recent decades have seen an increased adoption of mobile devices by the general public as well as the healthcare professionals [11,12]. Mobile devices have become commonplace in health care settings, leading to rapid growth in the development of medical software applications for these platforms [12,13]. Furthermore, mobile devices and applications have demonstrated compelling benefits, including increased access to point-of-care tools, enhanced clinical decision-making support, and improved patient outcomes [12,13]. However, the capabilities and the potential benefits of new technologies are most likely not optimally employed [12,13,14,15].

The reasons behind the gap between the guidelines and the real-world management of T2DM patients are not yet fully elucidated [10]. They can include inadequate education on novel treatment options and inefficient ways of communicating the advancements contained within the guidelines. Therefore, the aims of this study were (a) to estimate the familiarity of general practitioners from three European countries with different healthcare systems with novel type 2 diabetes mellitus (T2DM) treatment options; (b) to determine whether a digital tool can aid in their treatment decisions; and (c) to demonstrate that an evidence-based digital clinical support tool can be made using an existing digital platform.

## 2. Materials and Methods

This proof-of-concept study consisted of two parts: first, a simple online survey was conducted among general practitioners of three European countries; then a new digital tool that provides quick, evidence-based support for treatment of patients with T2DM was developed using an existing digital platform.

### 2.1. The Online Survey

The anonymous online survey consisted of seven multiple-choice questions (Table 1) that were conceptualized in cooperation between doctors with special interest in T2DM (authors) and a company that specializes in the development of clinical decision support tools (Mediately). The survey was designed to estimate the familiarity of general practitioners with novel T2DM treatment options and to determine whether they believe that a digital tool can aid in their T2DM treatment decisions (and therefore fulfill some of their unmet digital needs). First five questions of the survey enquired about the participants’ knowledge of novel treatments for T2DM (DPP-4 inhibitors, GLP-1 receptor agonists, and SGLT2 inhibitors) and the last two questions were designed to assess the eagerness of physicians to use a digital tool and to establish the most important features of such a tool, respectively. Demographic details and data on an individual physician’s level of expertise were not collected since an extensive analysis of the wide spectrum of potential causes for the possible results was out of scope of this study. Face validity of the questionnaire was ensured by collaboration between T2DM experts and information technology specialists; more advanced methods of questionnaire validation were not performed. All the questions and the supporting text were translated to responders’ local languages.

The survey included licensed general practitioners of three European countries (Italy, Czech Republic, and Slovenia), who were registered users of the Mediately application (Mediately Farmaci, Mediately Databáze léčiv and Mediately Register zdravil) and who had previously provided consent to be contacted for research purposes. In order to achieve a homogenous survey population, physicians of other specialties as well as trainees and residents were not included in the survey. The three countries with different healthcare systems were deemed to be representative of the region where Mediately currently operates (mainly Mediterranean and continental parts of South and Central Europe). The study was approved by the Slovenian National Medical Ethics Committee (approval No. 0120-300/2020/8).

In December 2021, an email with an explanation of the purpose of the questionnaire and a hyperlink to a web-based survey platform was sent to 5278 licensed general practitioners, users of the Mediately application. Based on Mediately’s previous experience with online surveys (the vast majority of recipients answered within a few days up to 1 week, and that those that did not answer during this time interval usually did not answer at a later time), the survey was active for 7 days, from 21 December 2021 to 27 December 2021. The results were collected using a dedicated survey platform and analyzed using one of the commonly used spreadsheet programs.

### 2.2. The Digital Clinical Decision Support Tool for Treatment of Patients with T2DM

Clinical decision support tools are systems that can link individual patient’s characteristics and physician’s findings with evidence-based data and thus assist in the healthcare decision-making process. The T2DM tool was conceptualized as an interactive digital step-by-step algorithm based on the treatment algorithm proposed by the 2019 ADA/EASD update on management of T2DM (2019 Update to: Management of Hyperglycemia in Type 2 Diabetes, 2018. A Consensus Report by the American Diabetes Association (ADA) and the European Association for the Study of Diabetes (EASD)) [2]. The tool was developed by the Slovenian company Mediately, which designed the Mediately application, a mobile and web-based application that provides drug information, clinical decision support tools, and continuing medical education content for healthcare professionals. The new T2DM digital tool became an integral part of the existing Mediately application.

The Mediately application is based on a uniform digital framework, which provides content to mobile and web-based platforms. The developers set up a certified quality management system for medical devices (ISO 13485:2016); the content, function and ergonomics of the tools are reviewed and tested by professionals of different fields, including medical doctors working in the company and several external experts. Most tools are also translated into users’ local languages by professional translators. The Mediately Clinical Tools collection is registered as a medical device and is available free of charge on mobile and web platforms in several European countries.

## 3. Results

### 3.1. The Familiarity of Physicians with Novel T2DM Treatments and Some of Their Digital Needs

The questionnaire designed to estimate the familiarity of general practitioners with novel T2DM treatment options and to appraise some of their digital needs pertaining to management of T2DM patients was completed by 129/5278 physicians (94 from Italy, 22 from Czech Republic and 13 from Slovenia; 2.44% response rate).

In general, only 30.7% (39/127) of general practitioners reported to be either very or extremely familiar with novel T2DM treatments. However, there were considerable variations among different countries: the Czech physicians were most familiar with the whole group of novel medications (63.6% either very or extremely familiar) while the Italian physicians more vaguely (only 22.3% were either very or extremely familiar). Slovenian and Italian physicians reported to have the best knowledge of SGLT2 inhibitors (46.2% and 26.6% either very or extremely familiar, respectively), whereas the Czech physicians were the ones most well-informed about the DPP-4 inhibitors and the GLP-1 receptor agonists (40.9% and 27.3% either very or extremely familiar, respectively).

Regarding the digital needs, the vast majority of physicians (82.8%, 106/128) reported that they would find very or extremely useful a digital tool to support their clinical decisions for treating T2DM patients, a kind of digital tool that, to our knowledge, did not exist prior to the development of our digital T2DM algorithm. The features that were considered to be of utmost importance were the compliance of the tool content with local guidelines, the ease of use, and the cost-free availability. Not all participants provided answers to all the questions of the survey; the question with the lowest number of valid responses was question No. 3 (familiarity with GLP-1 agonists) with 126 valid responses.

### 3.2. The Digital Clinical Decision Support Algorithm for Treatment of Patients with T2DM

**Figure 1:**
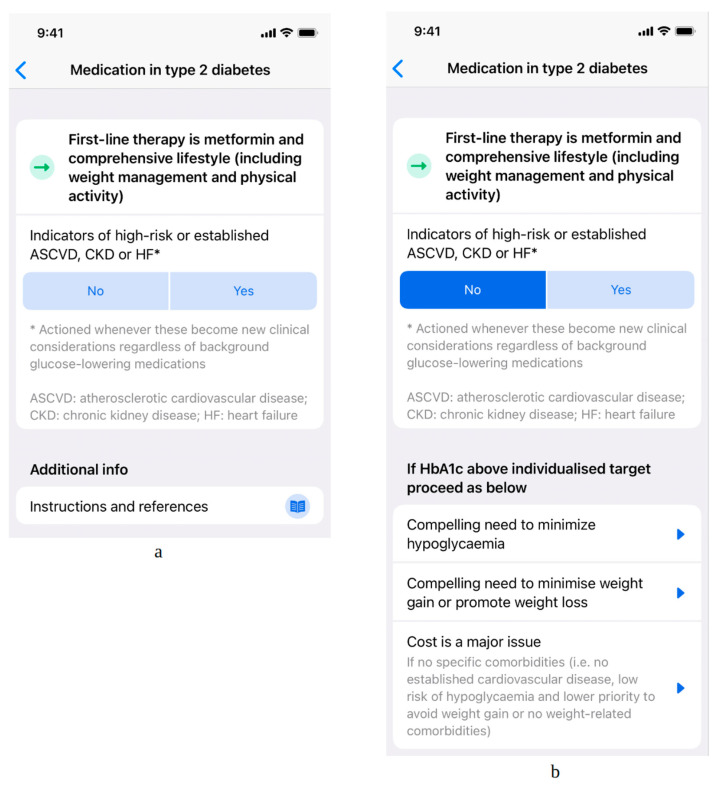
An illustrative usage scenario: (**a**) first-line therapy is suggested to the user, along with additional information in order to make a comorbidity-based treatment individualization; (**b**) different options for treatment individualization are suggested to the user, based on the presence or absence of specific comorbidities; additional information takes into account other concomitant factors, such as minimization of hypoglycemia, promotion of weight loss, and cost as a major issue. HbA1c: hemoglobin A1C (glycated hemoglobin).

**Figure 2:**
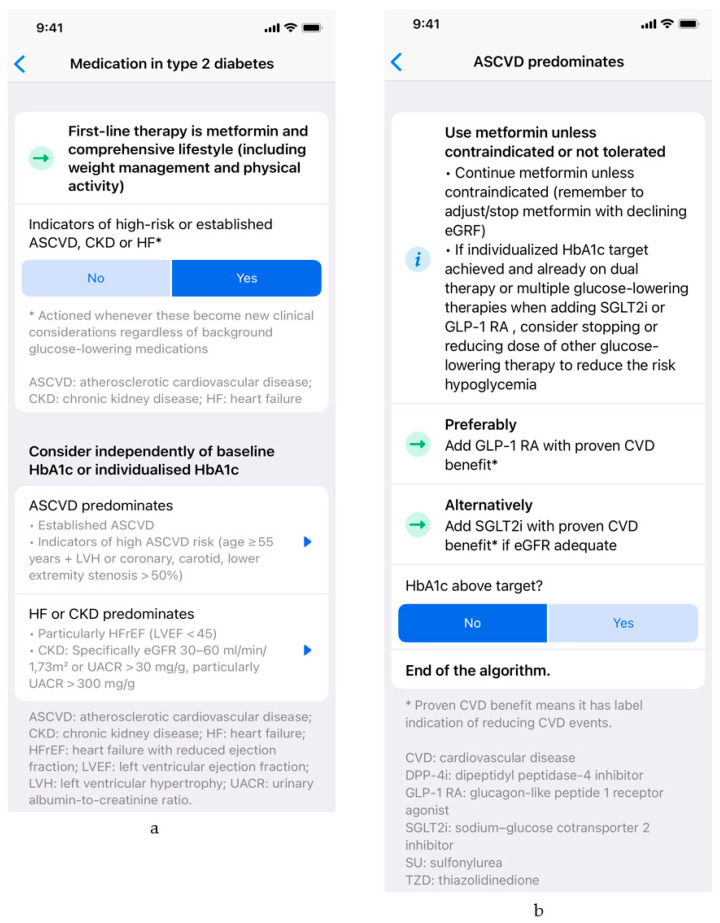
An illustrative usage scenario: (**a**) first-line therapy is suggested to the user, along with inquiry that enables comorbidity-based treatment individualization (predominating atherosclerotic cardiovascular disease, predominating heart failure, or chronic kidney disease); (**b**) specific recommendations for patients with predominating atherosclerotic cardiovascular disease are presented to the user. HbA1c: hemoglobin A1C (glycated hemoglobin); eGFR: estimated glomerular filtration rate.

The algorithm provides a digitalized version of main recommendations covered by the 2019 ADA/EASD update on management of T2DM (2019 Update to: Management of Hyperglycemia in Type 2 Diabetes, 2018. A Consensus Report by the American Diabetes Association (ADA) and the European Association for the Study of Diabetes (EASD)) [2]. It starts with the proposed first-line therapy and then branches into five major sections based on the presence or absence of comorbidities and individual patient characteristics. The digital algorithm therefore provides simple and easily accessible specific recommendations for first-line therapy of patients with T2DM, for groups of patients with atherosclerotic cardiovascular disease, chronic kidney disease or heart failure, and for patients with a compelling need to either minimize hypoglycemia, minimize weight gain, or reduce the cost of treatment. It features the characteristics that were reported to be most important to the polled physicians: it is based on European guidelines, it is easy to use and it is available free of charge. At the time of writing, the digital tool was already included in Czech and Slovenian versions of the Mediately Clinical Tools collection, available on mobile and web-based platforms. An illustrative usage scenario is depicted in Figure 1 and Figure 2.

## 4. Discussion

More than two-thirds of the polled physicians were at best moderately familiar with the new generation of T2DM medications that have now been available for about a decade. These groundbreaking medications provide some previously unseen and somewhat unexpected benefits; they address several important aspects of T2DM, the metabolic syndrome and a significant spectrum of the most substantial comorbidities [16]. In addition to their antihyperglycemic effects and concurrent lower risk of hypoglycemia, the GLP-1 receptor agonists and SGLT2 inhibitors demonstrated significant benefits for patients with atherosclerotic cardiovascular disease, heart failure, and chronic kidney disease [6,7]. For instance, liraglutide has several non-glycemic pleiotropic effects towards a reduction in the overall cardiovascular risk, such as the reduction in plasma total-cholesterol, triglyceride, and LDL-cholesterol concentrations as well as of carotid intima-media thickness [17]; interestingly, liraglutide has a direct anti-atherosclerotic effect by the reduction in atherogenic lipoproteins [18]. Furthermore, the GLP-1 receptor agonists and the SGLT2 inhibitors can help patients reduce weight while the DPP-4 inhibitors are weight-neutral [19,20,21,22]. Because of these critical added benefits, the addition of some of these novel medications is recommended even independently of glycemia levels or targets [3,5], and this emphasizes the cardiometabolic benefit of these novel anti-diabetic agents.

Reports dating back to 2014 have posited that digital tools can be used to address the challenges presented by modern management of chronic conditions [13]. A more recent literature review concluded that digital clinical decision-support tools have considerable potential to enhance access to care and quality of care, provided that the medical community rises to the challenge of modernizing its approach in order to capitalize on the opportunities of digitalization [12]. Indeed, four out of five physicians polled in our survey reported that they would clearly benefit from a digital clinical decision support tool, which indicates that at least some of the physicians’ digital needs are not sufficiently addressed. We believe we demonstrated that digital clinical decision support tools that can further address the physicians’ digital needs can be easily developed using existing digital platforms. Additionally, we showed that the features of the tool can be tailored according to physicians’ priorities. The developed T2DM digital tool brings the essential guideline content to most mobile phones and computers an easy-to-use, step-by-step format. It can therefore provide evidence-based content in a time-efficient manner to facilitate treatment decisions made by physicians treating T2DM patients.

We recognize that this study has certain limitations, including the relatively small number of participants to the survey, the lack of data on demographic characteristics of the participants, and the low response rate. Even though almost every European physician probably owns a mobile phone, the digital-only distribution of the questionnaire might have exposed the results to selection bias. Since the process of developing, updating, and validating a digital tool takes some time, the tool content is inevitably updated with some delay after the publication of new guidelines.

While response rates of <10% are not unusual for online surveys, we acknowledge that additional factors may have existed that resulted in an even lower response rate: the holiday season, lack of reminders and incentives for completion of the survey, the well-known time-constraints of general practitioners and the relatively short timeframe in which the online survey was active. However, in our experience, incentives and reminders do not consistently increase response rates and can appear disagreeable or annoying, especially to overburdened physicians. We also doubt that increasing the duration of the survey would majorly impact the response rate, potential participants usually become involved with online surveys in the first few days after receiving them or not at all. While it is possible that the low response rate might have impacted the validity of the survey to some extent, we believe that the results at the very least point to the existence of some unmet digital needs (that can demonstrably be fulfilled).

We wish to highlight that reliable digital tools have an even greater importance during the current coronavirus disease (COVID-19) pandemic. Indeed, different solutions of telemedicine have been implemented, and it is likely that daily usage of digital tools will be maintained during post-COVID-19 time for patient management [23]. This has a particular relevance for patients with T2DM [24], since their cardiometabolic complications have significantly increased in the last 2 years of COVID-19 pandemic due to the reduced access to healthcare facilities for regular/planned control visits [25]. We also observed that in many geographical areas, diabetes deteriorated more in patients with the poorest socio-economic situations, and this emphasizes the urgent need of a comprehensive and multidisciplinary approach for preventing cardiometabolic complications [26].

Bearing in mind the limitations of the current proof-of-concept study, we are convinced that novel digital tools can have a significant role in relieving some of the burden imposed on physicians by the constantly changing evidence-based medicine, and in optimizing the worldwide use of modern diagnostic and therapeutic procedures. More importantly, the use of reliable and effective novel digital tools can potentially improve the care of patients suffering from chronic diseases, including diabetes. We believe potential areas for future research include determining the reasons behind the low familiarity of general practitioners with novel T2DM medications and finding new innovative ways to improve digital clinical decision support systems.

## 5. Conclusions

The results of our online survey show that (a) the familiarity of general practitioners from Italy, Czechia, and Slovenia with novel T2DM treatment options is relatively low; (b) there is a need for a digital clinical decision support tool intended to facilitate treatment decisions in T2DM patients.

We demonstrated that an easy-to-use, evidence-based T2DM treatment clinical decision support tool can easily be developed using an existing digital platform, potentially contributing to improved patient care.

## Figures and Tables

**Table 1:** The anonymous online survey used.

Question 1	To which extent are you familiar with the efficacy and safety data of novel treatments for patients with type-2 diabetes?
Question 2	To which extent are you familiar with the efficacy and safety data on DPP-4 inhibitors?
Question 3	To which extent are you familiar with the efficacy and safety data on GLP-1 receptor agonists?
Question 4	To which extent are you familiar with the efficacy and safety data on SGLT2 inhibitors?
Question 5	To which extent are you familiar with the national prescription and reimbursement regulations for novel treatments?
Question 6	To which extent do you find useful a digital tool supporting your clinical practice with detailed summaries of the efficacy and safety data as well as the prescription and reimbursement criteria of novel treatments?
Question 7	In your opinion, which is the most prominent criteria for selecting a digital health care tool?

For questions 1 through 6, possible answers were: (a) not at all, (b) slightly, (c) moderately, (d) very, (e) extremely. For question 7, possible answers were: (a) content needs to be adapted to local guidelines, (b) ease of use, (c) validation of the digital tool, (d) whether the tool is free of charge.

## Data Availability

The data presented in this study are available on request from the corresponding author. The data are not publicly available due to privacy policy.
